# A Year in Review: JCAG’s Inaugural Editorial Fellowship

**DOI:** 10.1093/jcag/gwae037

**Published:** 2024-10-02

**Authors:** Jeffery M Venner

**Affiliations:** Section of Gastroenterology, Department of Internal Medicine, Max Rady College of Medicine, Rady Faculty of Health Sciences, University of Manitoba, Winnipeg, R3E 0W2, Canada

This past year I had the privilege of joining the *Journal of the Canadian Association of Gastroenterology* (JCAG) as its inaugural Editorial Fellow. The position was developed with the goal of providing an interested trainee of the Canadian Association of Gastroenterology (CAG) experience in the peer-review and publishing process, as well as exposure to the inner workings of Canada’s premiere gastroenterology journal. Given my career goal of becoming a clinician-scientist, I was excited and honoured to be awarded this position. The experience was invaluable.

Like most medical residents, gastroenterology trainees have varying degrees of research experience. However, the opportunity to learn about the scientific publishing industry is certainly unique. Through attendance at monthly meetings with the JCAG’s publisher (Oxford University Press), I was able to gain an understanding of the editorial and publishing side of research from both an administrative and academic perspective. I was exposed to many challenges that all academic journals undoubtedly face, for example: the process of being listed by various indexing services and applying for an impact factor, budgeting and financial restraints, advertising, and partaking in a revamp of the JCAG’s website and print design. I experienced the never-ending challenge that most academic journals encounter with attracting and rewarding editors and manuscript peer reviewers, who all volunteer their time and expertise.

One of the most rewarding components of the JCAG Editorial Fellowship was the experience of reviewing manuscripts and taking on an Associate Editor position. I honed communication skills, including the process of giving thoughtful and constructive feedback. I also noted how individual reviewers each bring different experiences and strengths to the peer-review process. The peer-review process is a tool for strengthening research. It allows us to give back to the research community through the promotion of quality research—a process that undoubtedly has criticisms but is there to aid in the advancement of knowledge, support innovation, and ultimately benefit our patients.

Through all of this, I gained insight into new and emerging research trends and identified unmet needs in today’s academic and clinical gastroenterology environment. Partaking in the review process allows one to see the latest research prior to publication, which can be very exciting. This sharpened my curiosity and tenacity, both vital skills in research. However, arguably most importantly, these experiences have the potential to positively influence patient care through my practice as a gastroenterologist.

It was a privilege to receive attentive and insightful mentorship from the Editor-in-Chief (Dr Eric Benchimol) and Associate Editors (Dr Talat Bessissow and Dr Sanjay Murthy) of JCAG, who were not only invested in the success of the new position but also my personal success as well, an honour for any trainee. This position was designed with the aim of supporting and encouraging the development of budding academic gastroenterologists, and I feel strongly that the combination of mentorship and experience has done so and will continue to contribute to the education and career development of future JCAG Editorial Fellows. I am grateful for this opportunity, and I am excited to continue to work with the gastroenterology community in Canada and abroad.

## Supplementary material

Supplementary material is available at *Journal of the Canadian Association of Gastroenterology* online.

gwae037_suppl_Supplementary_Material

## Data Availability

There are no data associated with this editorial article.

